# Salt-Sensitive Hypertension: Mediation by Salt-Induced Hypervolemia and Phosphate-Induced Vascular Calcification

**DOI:** 10.1177/11795468231158206

**Published:** 2023-07-06

**Authors:** Ronald B Brown

**Affiliations:** School of Public Health Sciences, University of Waterloo, Waterloo, ON, Canada

**Keywords:** Salt-sensitive hypertension, hypervolemia, vascular calcification, phosphate toxicity, sodium chloride, elastin, hyperphosphatemia

## Abstract

Preventing hypertension by restricting dietary salt intake, sodium chloride, is well established in public health policy, but a pathophysiological mechanism has yet to explain the controversial clinical finding that some individuals have a greater risk of hypertension from exposure to salt intake, termed salt-sensitive hypertension. The present perspective paper synthesizes interdisciplinary findings from the research literature and offers novel insights proposing that the pathogenesis of salt-sensitive hypertension is mediated by interaction of salt-induced hypervolemia and phosphate-induced vascular calcification. Arterial stiffness and blood pressure increase as calcification in the vascular media layer reduces arterial elasticity, preventing arteries from expanding to accommodate extracellular fluid overload in hypervolemia related to salt intake. Furthermore, phosphate has been found to be a direct inducer of vascular calcification. Reduction of dietary phosphate may help reduce salt-sensitive hypertension by lowering the prevalence and progression of vascular calcification. Further research should investigate the correlation of vascular calcification with salt-sensitive hypertension, and public health recommendations to prevent hypertension should encourage reductions of both sodium-induced hypervolemia and phosphate-induced vascular calcification.

## GRAPHICAL ABSTRACT


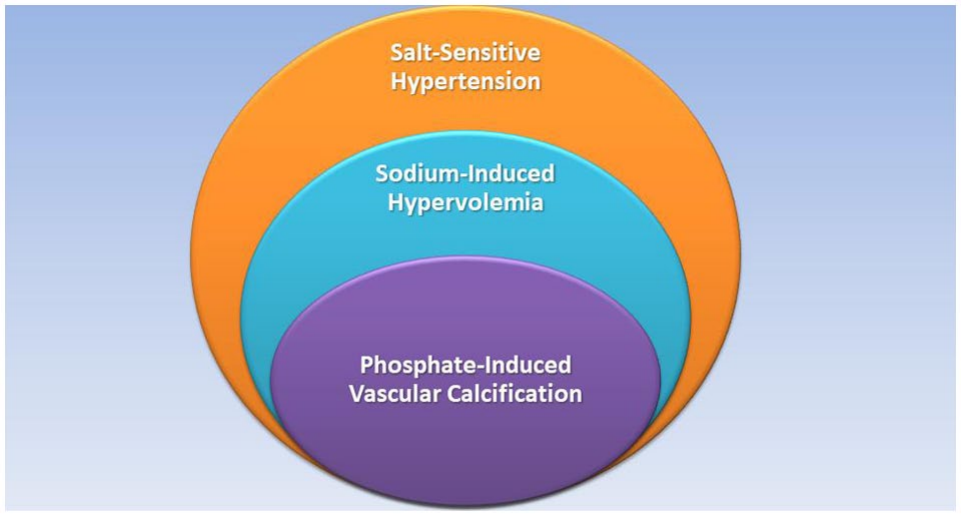


## Introduction

World Health Organization (WHO)^1^ recently published key facts about hypertension, defined as the effect on the arterial walls from high blood pressure of ⩾140 mmHg systolic pressure during heartbeat contraction and ⩾90 mmHg diastolic pressure during heartbeat relaxation. Hypertension is a major cause of premature death, and current worldwide prevalence of hypertension is 1.28 billion adults between the ages 30 and 79 years. WHO’s global target “is to reduce the prevalence of hypertension by 33% between 2010 and 2030.”

Hypertension pathogenesis involves many complex interactions between environmental, pathophysiological, and genetic factors that affect multiple organ systems.^2^ The close association of hypertension with dietary sodium chloride, common table salt, is “widely recognized and supported by several studies.”^3^ WHO listed “excessive salt consumption” as a modifiable risk factor for hypertension and recommended reducing salt consumption to 5 g a day as a public health nutrition policy. However, other authors argued that “individual effects of salt intake on blood pressure are highly variable, leading to contentious debates about public policy,” and the authors noted that “some people are especially sensitive to salt intake, and with a typical U.S. diet, hypertension develops in those people.”^4^ Accordingly, the PURE study found that systolic blood pressure in older individuals and people with hypertension increased by 2.11 mmHg for every increased gram of dietary sodium intake, but “there was little effect in those without hypertension or those <55 years.”^5^ Similarly, a recent controlled feeding study found that high amounts of salt intake compared to medium and low salt intake did not increase peripheral blood pressure variability in healthy, young, normotensive adults.^6^ More research is needed to resolve this clinical controversy by clarifying the pathogenic mechanisms underlying salt-sensitive hypertension:

“Despite extensive research, the pathogenesis of salt-sensitive hypertension is still unclear and tremendously challenged by its multifactorial etiology, complicated genetic influences, and the unavailability of a diagnostic tool.”^7^

## Method

The present perspective paper investigated mediating factors underlying the variable association of sodium and blood pressure in salt-sensitive hypertension observed within the general population. A grounded theory method was used to add rigor and objectivity to the literature review in this perspective paper.^8^ Interdisciplinary findings from across the research literature on dysregulated sodium metabolism, hypertension, vascular calcification, vascular elasticity, and dysregulated phosphate metabolism were iteratively compared and synthesized into theoretical concepts and themes. An evidence-based theory was formed that proposes a novel pathophysiological mechanism in which the interaction of phosphate-induced vascular calcification and sodium-induced hypervolemia mediate salt-sensitive hypertension.

### Salt sensitivity and hypervolemia

The American Heart Association (AHA) defines salt sensitivity in humans as a physiological trait in which blood pressure “of some members of the population exhibits changes parallel to changes in salt intake.”^9^ In earlier investigations, Weinberger^10^ found salt sensitivity in 26% of normotensive individuals and in 51% of individuals with hypertension.

Referring to Guyton’s foundational research positing that blood pressure equals pulse volume times peripheral resistances, researchers noted that pulse volume rises as dietary salt increases intravascular fluid.^11^ For example, saline fluids containing salt are used by clinicians “to increase intravascular fluid volume.”^12^ However, excessive sodium intake leads to fluid overload and expanded volume of extracellular fluid (ECF) in hypervolemia.^13^

“Hypervolemia refers to expansion of ECF volume, which varies, even in normal individuals, with dietary sodium intake. . .In most individuals, this increase in ECF volume is not clinically detectable and does not have pathologic consequences. In some individuals, however, this upward shift in ECF volume increases systemic arterial blood pressure. When the sodium surfeit expands the ECF volume beyond the range necessary for the adjustment needed to restore sodium balance, a state of pathologic hypervolemia ensues.”^14^

The kidneys normally adjust sodium balance through urinary excretion, but increased intake of sodium chloride is associated with impaired kidney function,^15^ potentially leading to sodium imbalances that contribute to hypervolemia. Additionally, hypertonic dehydration caused by sodium chloride intake may trigger activation of the Renin-Angiotensin-Aldosterone System (RAAS) which increases reabsorption of sodium and fluid in the kidneys,^16^ further contributing to salt and fluid retention in hypervolemia. Importantly, “evidence has accumulated showing that impairment in vascular function may play a relevant role in salt-sensitive hypertension.”^3^ Factors that impair vascular function and increase peripheral vascular resistance may determine whether blood pressure exhibits changes parallel to increased pulse volume from sodium-induced hypervolemia, consistent with Guyton’s mechanism for blood pressure. One such resistance factor in vascular function is vascular calcification, discussed in the following text.

### Salt sensitivity and vascular calcification

Salt sensitivity is more common in people with chronic kidney disease (CKD), insulin resistance, females, the elderly, and Afro-Americans.^11^ Coincidently, vascular calcification (VC), defined as “the pathological deposition of mineral in the vascular system,”^17^ is comorbid with salt sensitivity in most of these segments of the population. For example, vascular calcification in patients with CKD is a major cause of cardiovascular morbidity and mortality,^18^ and VC in CKD “correlates with both lower estimated glomerular filtration rate (eGFR) and disordered bone mineral metabolism.”^19^ Insulin resistance was found to predict arterial calcification in men,^20^ and middle-aged Afro American women were found to have greater coronary artery calcification (CAC).^21^ These examples of comorbidities with salt sensitivity suggest that vascular calcification is a potential factor associated with peripheral vascular resistance in salt-sensitive hypertension.

**Figure 1.**
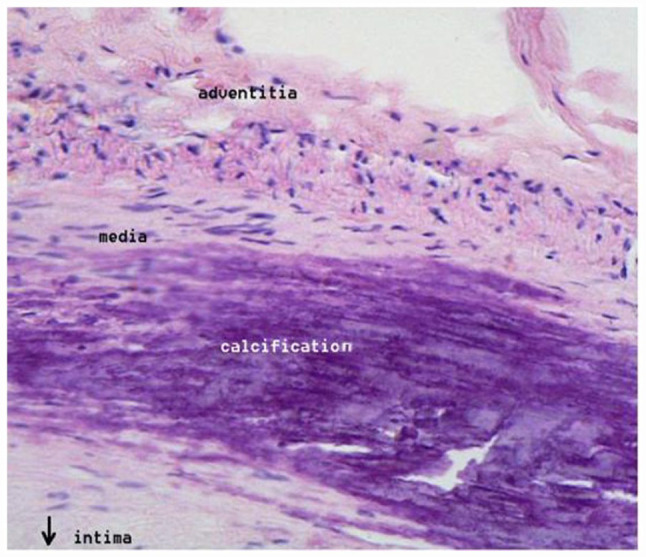
Medial calcification. Public Domain. Retrieved December 22, 2022 from https://commons.wikimedia.org/w/index.php?curid=446171].

Mineral deposits in VC consist of hydroxyapatite, normally found in bone, and calcification occurs in the medial vascular layer, appearing as “organized mineral deposition along the elastic lamellae of the arterial wall media” which decreases media elasticity and increases arterial stiffening.^22^Figure 1 shows calcification in the media vascular layer, which lies between the outermost adventitia and innermost intima layers of arteries.

Normally, elastin in arteries “provides reversible extensibility during cyclic loading of the cardiac cycle,”^23^ and the elastic wall of arteries distends to accommodate higher pulse volume loads, thus mitigating increases in intravascular pressure. “The elastic nature of the aorta, for example, allows the vessel to distend to accommodate the considerable increase in blood volume when the heart goes into systole.”^24^ Summarizing the function of elastin in the vascular system:

“The aorta, pulmonary artery, and large distributing arteries are distended rapidly during ventricular ejection, transiently accommodating 50% or more of the stroke volume. These vessels then retract during diastole. Because of these dimensional changes, the viscoelastic properties of the walls of these large vessels are a factor determining instantaneous arterial pressure.”^25^

Arterial elasticity likely accounts for the observation that many people, especially young people, experience little change in blood pressure from sodium-induced hypervolemia, provided arteries retain their elasticity to accommodate increases in pulse/stroke volume. However, gradually over time, the interaction of sodium-induced hypervolemia with increasing inelasticity and stiffening of the peripheral vascular system due to VC could account for salt-sensitive hypertension. The proposed interaction of hypervolemia and VC might be blunted either by lowering hypervolemia through reduced salt intake, and/or by lowering modifiable risk factors associated with vascular calcification, such as dietary phosphate overload, discussed next.

### Phosphate-induced vascular calcification

Phosphorus, an essential mineral in the body found in the form of phosphate (PO_4_), is regulated by a network of hormones released by the kidneys, bone, intestines, and parathyroid glands.^26^ Dysregulation of phosphate metabolism can raise serum phosphate levels, causing hyperphosphatemia, and produce toxic accumulations of phosphate in the body, termed phosphate toxicity, which adversely affects almost every major organ system. In a study finding that overload of dietary inorganic phosphate (Pi) in rats directly induced hyperphosphatemia and aortic medial calcification, the authors wrote:

“Hyperphosphatemia induces VC via trans differentiation of vascular smooth muscle cells (VSMCs) into osteoblast-like cells and via apoptosis of VSMCs, which is triggered by Pi entry into cells through Pi transporter (Pit)-1.”^27^

The authors suggested that phosphate-induced vascular calcification could be mitigated through dietary phosphate interventions, but more research is needed in this area.

Other authors found that hyperphosphatemia stimulated expression of inflammatory cytokines by activating the Toll-like receptor 4 (TLR4)/nuclear transcription factor κB (NF-κB) signaling pathway for inflammation, and increased the expression of both Runt-related transcription factor 2 (Runx2) and bone morphogenetic protein-2 (BMP2), “considered to be an important molecular marker of VC.”^28^ Phosphate also unites with calcium to form calcium phosphate in VC mineralization, and “collagenization of the media and accumulation of calcium and phosphate in elastic fibers progressively occurs with aging.”^29^ Importantly, elastin-degrading enzymes, elastases, include matrix metalloproteinases (MMPs),^30^ and phosphate-induced expression of MMP2 and MMP9 in VSMC calcification promotes elastin degeneration.^31^

In chronic kidney disease, which is also associated with salt sensitivity, dysregulated calcium and phosphate metabolism accounts for bone mineral disorders and lower eGFR^32^ and “drives vascular calcification” in CKD patients.^33^ High serum phosphate levels and age of patients receiving peritoneal dialysis were independent risk factors for progression of CAC.^34^

In addition, secondary hyperparathyroidism (SHPT) is associated with high serum Pi and VC,^35^ and hypertension is very frequently associated with SHPT in hemodialysis patients,^36^ potentially mediated by VC. Moreover, parathyroidectomy lowers VC,^37^ which likely explains why blood pressure in hypertensive hemodialysis patients dropped over approximately 9 months following parathyroidectomy in an earlier study,^38^ and similarly in a more recent study.^39^ Fibroblast growth factor 23 (FGF-23) from bone is also associated with high serum Pi, and incidence and prevalence of hypertension is associated with high FGF-23 in community-living older adults.^40^ Furthermore, FGF-23 in hypertension is also associated with sodium retention and increased plasma volume.^41^

A recent cross-sectional study using the Ankle-Brachial Index (ABI) to evaluate VC within the general population found a high prevalence of vascular calcification among people aged 30 to 70 years.^42^ Unfortunately, a medical therapy to effectively treat CAC “has yet to be established; therefore a greater understanding of the factors that induce calcification is needed to develop appropriate therapeutic strategies.”^43^ Based on findings from epidemiological and clinical studies and experimental models, “phosphate is now accepted as a major direct inducer of VC.”^44^ “Avoiding phosphate loading” is part of a clinical approach in non-dialysis CKD to lower VC incidence and progression.^45^ Additionally, higher serum phosphate levels within the normal range in healthy young adults in the study of Coronary Artery Risk Development in Young Adults (CARDIA) were associated with a 52% higher risk of coronary artery calcium in comparison with lower serum phosphate levels.^46^

Of public health relevance, mean dietary phosphate intake among U.S. adults is much higher than the Dietary Reference Intake of 700 mg per day, with all-cause mortality increasing at >1400 mg of phosphate.^47^ Therapeutic interventions targeting reduced salt-sensitive hypertension may profit from including modification of dietary phosphate intake along with reduced salt intake. Restricted dietary phosphate intake, 800 to 1000 mg/day recommended by the U.S. National Kidney Foundation’s Kidney Disease Outcomes Quality Initiative, is already in use for CKD.^48^ Future research should also investigate the correlation of salt-sensitive hypertension in individuals with vascular calcification.

**Figure 2.**
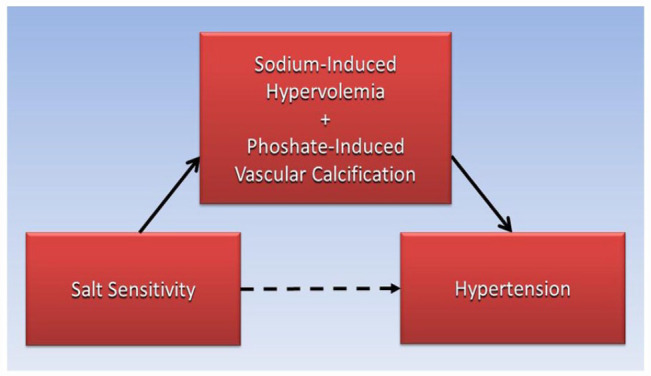
Proposed mediation of salt sensitivity associated with hypertension through the interaction of sodium-induced hypervolemia and phosphate-induced calcification.

Figure 2 is a directed acyclic graph proposing that the association of salt sensitivity with hypertension, the dashed arrow, is mediated by the interaction of sodium-induced hypervolemia and phosphate-induced vascular calcification within the causative pathway, the solid arrows.

## Conclusion

The pathogenesis of salt-sensitive hypertension remains unclear, and the clinical controversy affecting public health policy remains unresolved, despite much research. Novel insights provided in the present perspective paper propose that the association of salt sensitivity with hypertension is mediated by interaction of sodium-induced hypervolemia and phosphate-induced vascular calcification. Phosphate has been found to be a direct inducer of calcification. Medial calcification reduces arterial elasticity and increases arterial stiffness and blood pressure, preventing arteries from expanding to accommodate extracellular fluid overload in hypervolemia. Salt intake increases extracellular fluid, and salt reduction is often recommended to reduce hypertension. However, additional reductions of dietary phosphate may help reduce salt-sensitive hypertension by lowering the prevalence and progression of vascular calcification. Further research should investigate the correlation of vascular calcification with salt-sensitive hypertension.
